# Degradation of Poly(3-hydroxybutyrate-co-3-hydroxyvalerate) Reinforced with Regenerated Cellulose Fibers

**DOI:** 10.3390/polym16142070

**Published:** 2024-07-19

**Authors:** Michael Seitz, Rainer Rihm, Christian Bonten

**Affiliations:** 1 Institut für Kunststofftechnik, University of Stuttgart, Pfaffenwaldring 32, 70569 Stuttgart, Germany; 2 Fraunhofer Institute for Applied Polymer Research IAP, Geiselbergstraße 69, 14476 Potsdam, Germany

**Keywords:** PHBV, regenerated cellulose fibers, accelerated aging, artificial weathering, degradation, hydrolysis

## Abstract

PHBV is a promising plastic for replacing conventional petroleum-based plastics in the future. However, the mechanical properties of PHBV are too low for use in high-stress applications and the degradation of the polymer limits possible applications. In this work, the mechanical properties were, therefore, increased using bio-based regenerated cellulose fibers and degradation processes of the PHBV-RCF composites were detected in accelerated aging tests under various environmental conditions. Mechanical, optical, rheological and thermal analysis methods were used for this characterization. The fibers significantly increased the mechanical properties, in particular the impact strength. Different degradation mechanisms were identified. UV radiation caused the test specimens to fade significantly, but no reduction in mechanical properties was observed. After storage in water and in aqueous solutions, the mechanical properties of the compounds were significantly reduced. The reason for this was assumed to be hydrolytic degradation catalyzed by higher temperatures. The hydrolytic degradation of PHBV was mainly caused by erosion from the test specimen surface. By exposing the regenerated cellulose fibers, this effect could now also be visually verified. For the use of regenerated cellulose fiber-reinforced PHBV in more durable applications, the aging mechanisms that occur must be prevented in the future through the use of stabilizers.

## 1. Introduction

Conventional plastics are based on the limited resource of crude oil and, therefore, contribute to the release of fossil carbon into the atmosphere. In the future, biologically based plastics can help protect the environment and reduce dependence on fossil resources. The growing awareness of the environment and sustainability has led to the market for bioplastics currently experiencing highly dynamic development [1]. 

**Figure 1:**
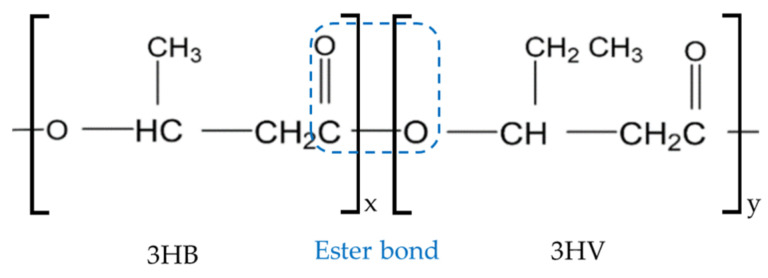
Molecular structure of poly-3-hydroxybutyrate-co-3-hydroxyvalerate.

Promising representatives of these bioplastics are polyhydroxyalkanoates (PHAs). PHAs are bio-based and biodegradable polyesters that are suitable for numerous applications due to their wide range of properties [2,3,4]. Poly(3-hydroxybutyrate-co-3-hydroxyvalerate) (PHBV) is one of the most studied and used PHAs [5,6]. The properties of PHBV are highly dependent on the molecular structure, which is shown in Figure 1 [4].

PHBV is composed of 3-hydroxybutyrate (3HB) and 3-hydroxyvalerate (3HV) building blocks, each with three carbon atoms in the main chain of the polymer. 3HB and 3HV differ only in the methyl or ethyl group. The monomers are linked via an ester bond [7]. 

PHBV has a high crystallinity, which decreases as the 3HV content increases. As a result, PHBV has a higher elongation at break and impact strength compared to P(3HB) [4,8]. However, the disadvantages of PHBV continue to be its low mechanical properties and the aging mechanisms that occur, which weaken the material over time. The chemical degradation of PHBV mainly follows five different reaction mechanisms. These reaction mechanisms are autocatalytic radical reactions, hydrolysis, photochemical reactions Norrish type I and II and, to a lesser extent, cross-linking reactions [9,10]. The breakage of the polymer chains results in a decrease in molecular weight. This leads to a decrease in melt viscosity. The extensive changes caused by chemical and physical aging also change the thermal properties as well as the degree of crystallization of the PHBV and usually result in a reduction in the mechanical properties and the embrittlement of the material [9,11,12].

Due to this susceptibility to aging, the moderate mechanical properties in combination with the excellent biocompatibility and biodegradability, PHBV is currently mainly used in short-lived disposable products such as food packaging [2,13]. 

To make PHA suitable for applications that are exposed to higher mechanical loads, fillers and reinforcing materials can be used [4]. Natural reinforcing materials such as nettle fibers, pine cone flour and walnut shell flour are used for this purpose [14]. In some works, natural fibers made from maple, bamboo or straw or nanofillers such as graphene or nanomaterials are utilized [5]. Several studies are investigating the use of cellulose in the form of fibers or powder to modify PHBV [6,15,16]. All these approaches are only able to increase the mechanical properties slightly or the bio-based character is lost through the use of fillers.

Cellulose regenerated fibers (RCFs) are a bio-based reinforcing material that has the potential to increase the mechanical properties of plastics [17,18]. For example, the use of RCFs has increased the strength of PLA to up to 120 MPa and the impact strength to up to 120 kJ/m^2^ [17]. To produce these fibers, the cellulose is dissolved in a multi-stage chemical process, then pressed through a nozzle into a precipitation bath and regenerated into fiber strands. Unlike conventional natural fibers, RCFs have reproducible material properties due to the production process [19,20] (p. 859). So far, RCFs have mainly been used in polyolefins [17,18,21,22,23,24,25] or PLA [23,26,27,28,29] as matrix materials due to the relatively low processing temperatures and, thus, lower thermal fiber damage. In some studies, however, higher melting polymers such as biopolyamides [30,31,32] with melting temperatures above 200 °C are also used. Due to the low processing temperatures of PHAs, it can be used as a matrix for RCFs, which could result in completely biobased and biodegradable compounds. The few studies carried out to date show that the very brittle PHAs became significantly tougher through the use of the fibers and further material properties could be improved [17,33]. However, in addition to the initial properties of the compounds, the influence of various environmental conditions and the resulting aging processes are also essential for their use in products. So far, however, there have been no studies on the resistance of compounds made from RCF-reinforced PHAs.

## 2. Materials and Methods

### 2.1. Fiber and Matrix

A PHBV EnmatY1000P from TianAn Biologic Materials Co. Ltd., Ningbo, China was used for the investigations. This is a copolymer with an HB:HV ratio of 98:2. The PHBV contains the nucleating agent boron nitride, which results in a high degree of crystallization, a fine crystal structure and rapid secondary crystallization for PHAs [34]. Preliminary tests confirm that the degree of crystallization and the mechanical properties hardly change 10 days after the test specimens were produced. Nevertheless, all tests were carried out after 21 days at the earliest. 

The regenerated cellulose fiber CR 2440 dtex Z100 from Cordenka, Obernburg am Main, Germany, was used as reinforcing material. The fiber has a strength of 830 MPa, a modulus of elasticity of 20 GPa and an elongation at break of 13%. Before processing, the fibers were in the form of continuous filaments.

### 2.2. Double Pultrusion

Compounds were manufactured in two steps using the double pultrusion process. The matrix material was dried overnight at 60 °C in the presence of silica gel in a nitrogen atmosphere. In the first production step, six strands of the fiber material were endlessly coated with the matrix material. A single-screw extruder from Dr. Collin, Ebersberg, Germany, was used for this purpose. The haul-off speed was adjusted so that the ratio of fiber and matrix was 20:80. The Haake haul-off device used was set to 400 scale parts. This resulted in a strand weight of 7.3 g/m. The five heating zones of the extruder were heated to 185 °C from the feed to the die (60 °C, 170 °C, 180 °C, 180 °C and 180 °C). A melt temperature of 172 °C was determined at a pressure of 42 bar. The extracted strand was cooled in a water bath and shortened to pellet lengths of 5 mm.

Before homogenization in a second extrusion step, the material was again dried overnight. A co-rotating twin-screw extruder from Berstorff, Hannover, Germany, with a screw diameter of 24 mm was used to homogenize the fiber and matrix material. Dosing was carried out with a spiral screw without a core in a Brabender dosing unit. The extruder was operated at a screw speed of 60 rpm and a torque of 50 Nm. The 8 zones of the extruder were set uniformly from 150 °C to 160 °C. The die had a temperature of 170 °C. A melt temperature of 181 °C was measured in zone 7. A vacuum of 28 mbar was applied to zone 7 via a dome in order to remove any possible condensate from the extruder. The used Scheer pelletizer was set to 204 scale parts and the resulting strand was shortened to 5 mm long pellets.

### 2.3. Injection Molding

Type 1A test specimens according to DIN EN ISO 3167 [35] were manufactured using a Boy injection molding machine (type 25), Neustadt-Fernthal, Germany. The granulate, which was dried overnight at 50 °C in a vacuum oven, was injected at an injection speed of 40 scale parts. The heating zones of the injection molding machine were set to 150 °C, 160 °C and 165 °C. The temperature of the nozzle delivered the best results at 170 °C. An injection pressure of 350 bar and a holding pressure of 300 bar were selected. The cooling time was 17 s at a mold temperature of 50 °C. 

### 2.4. Accelerated Aging

To investigate the resistance and the degradation mechanisms, accelerated aging tests were carried out under various environmental conditions and compared to tests after a standard climate (SC) storage at 23 °C and 50% relative humidity as a reference. Apart from the 21-day accelerated aging tests, all test specimens were stored in the SC from the time of specimen production until characterization.

Table 1 shows the storage conditions carried out, the parameters used and the media in which the test specimens were stored.

For temperature storage, the temperature was set up to 70 °C. Freezing storage at −18 °C was carried out to investigate a possible aging effect at low temperatures. A radiation density of W = 1000 W/m^2^ was used for accelerated aging under UV radiation. Humidity storage at 90% relative humidity and water storage in distilled water were also carried out. Further water storage was carried out at an elevated temperature of 70 °C to investigate possible hygrothermal degradation. The resistance of the compounds to acids and bases was investigated by storing them in citric acid and soda lye. Solutions with pH values of 2 were prepared with distilled water by adding citric acid and 12 by adding sodium hydroxide.

### 2.5. Tensile Test

Tensile tests were carried out with type 1A specimens in accordance with DIN EN ISO 527 [36]. A Zwick 1455 tensile testing machine from ZwickRoell GmbH & Co. KG, Ulm, Germany, with a pneumatic clamping device was used. The tensile tests were carried out at a testing speed of 5 mm/min. Young’s modulus was determined at 1 mm/min. In addition to the stress–strain diagrams, the tensile strength, Young’s modulus and elongation at break were determined in the tests. The number of specimens tested per material combination was seven.

### 2.6. Impact Test

Charpy impact bending tests were carried out in accordance with the DIN EN ISO 179-1 standard [37]. A pendulum impact tester 5102 from Zwick-Roell, Ulm, Germany, with a 15 J pendulum was used for the tests. The unnotched test specimens were prepared from the manufactured type 1A test specimens by sawing off the shoulders. Ten repeat measurements were carried out in each case.

### 2.7. Scanning Electron Microscopy

The fracture surfaces of the test specimens broken in the impact test were examined using Scanning Electron Microscopy (SEM). This allowed the fiber–matrix adhesion to be quantitatively evaluated. A CamScan MV2300 from EOS, Ottawa, ON, Canada, was used for this purpose. An accelerating voltage of 10 kW and magnifications of up to a factor of 1000 were used. Before the examination, the fracture surfaces were coated with gold.

### 2.8. Color Tests

Color measurements were carried out on weathered compounds to detect any aging-related discoloration. A CM-700d colorimeter from Konica Minolta, Chiyoda, Japan, was used. The results of the measurements are given in the Lab color space, which describes all colors that can be perceived by the human eye. The three-dimensional color space consists of the color axes a* and b* as well as the perpendicular brightness value L*. The L* axis ranges from the value 0 (black) to the value 100 (white). The color axes range from the values −128 to 127, which corresponds to the colors from green to red on the a* scale and blue to yellow on the b* scale. All measurements were carried out on five test specimens.

### 2.9. Macroscopic Investigations

In order to detect changes on the surface, macroscopic images were taken using a Wild M420 type 246634 from Heerbrugg, Balgach, Switzerland. The images after accelerated aging under UV radiation were taken on the side of the test specimen facing the UV lamp.

### 2.10. Weight Measurements

The weight of the test specimens was measured before and directly after all accelerated aging tests and after reconditioning. For reconditioning, the samples were stored for 21 days under standard climate at 23 °C and a relative humidity of 50%. A Mettler Toledo Excellence Plus XP2003S from Mettler Toledo, Columbus, OH, USA, was used for the weight measurements. Five test specimens were weighed in each case.

### 2.11. Rheological Investigations

To characterize possible degradation mechanisms on the polymer, rheological investigations were carried out using a plate–plate rheometer of the type DHR 2 Discovery Hybrid Rheometer from TA Instruments, Eschborn, Germany. The frequency sweeps were performed at 180 °C. Three measurements were taken in each case. The measurements were carried out on unreinforced PHBV test specimens, as the possible degradation of the polymer was to be investigated and fiber-reinforced materials can lead to inaccurate measurements.

## 3. Results and Discussion

### 3.1. Preliminary Investigations of the Compounds

**Figure 2:**
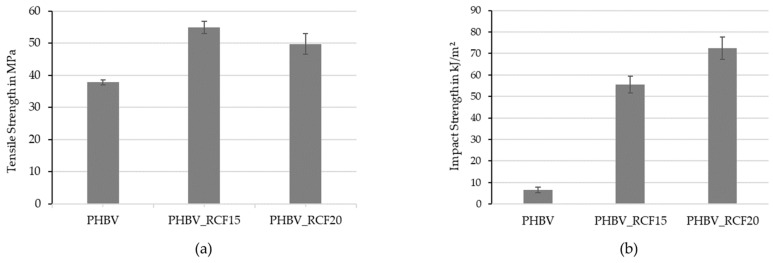
Tensile strength (**a**) and impact strength (**b**) of PHBV-CR compounds with different fiber contents.

Compounds with different fiber contents were produced and characterized in preliminary tests. The determined tensile strengths and impact strengths of the unreinforced PHBV and the compounds are shown in Figure 2. The tensile strength of the unreinforced PHBV of 37.81 MPa was increased by 45.2% to 54.9 MPa by adding 15 wt.% RCFs. The tensile strength was not increased any further by increasing the fiber content. The brittle behavior of the PHBV could be significantly improved using RCFs. The low impact strength of the unreinforced PHBV of 6.6 kJ/m^2^ was increased by a factor of 10 to 55.6 kJ/m^2^ with 15 wt.% RCFs and 72.46 kJ/m^2^ with a fiber content of 20 wt.%.

**Figure 3:**
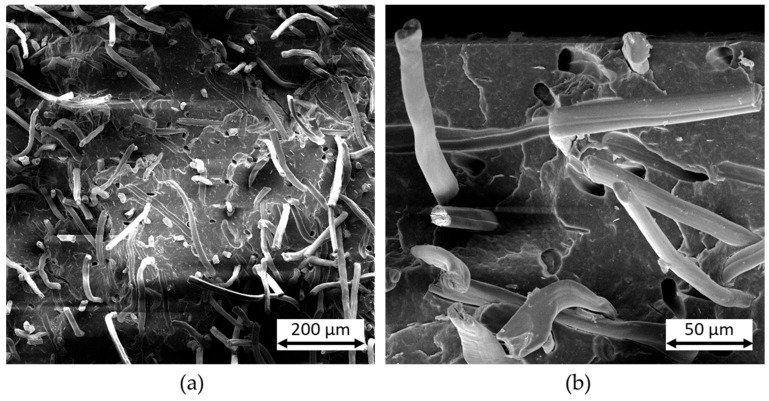
SEM images of an unweathered compound with 20 wt.% fiber content after impact test. (**a**) Many intact fibers after breakage (**b**) long fiber pull-outs and remaining fiber channels in the matrix.

The reason for this great increase in toughness can be seen in Figure 3. SEM images of the fracture surfaces after an impact test are shown. In the images, many long and intact fibers can still be seen. A large proportion of the fibers were pulled out of the matrix during the impact load without breaking. This fiber extraction is also clearly visible in the matrix due to the numerous holes present. No matrix material adheres to the fibers and smooth pull-out channels are created. The strength is only moderately increased due to the weak fiber–matrix adhesion. Due to the high energy absorption through friction when the fibers are pulled out, this results in a high increase in the impact strength of the compounds. This effect was also observed and described in different studies [18,21,32,38]. Due to the increase in toughness, PHBV-RCF compounds with a fiber content of 20 wt.% were used for further tests.

### 3.2. Color Testing and Macroscopy

**Figure 4:**
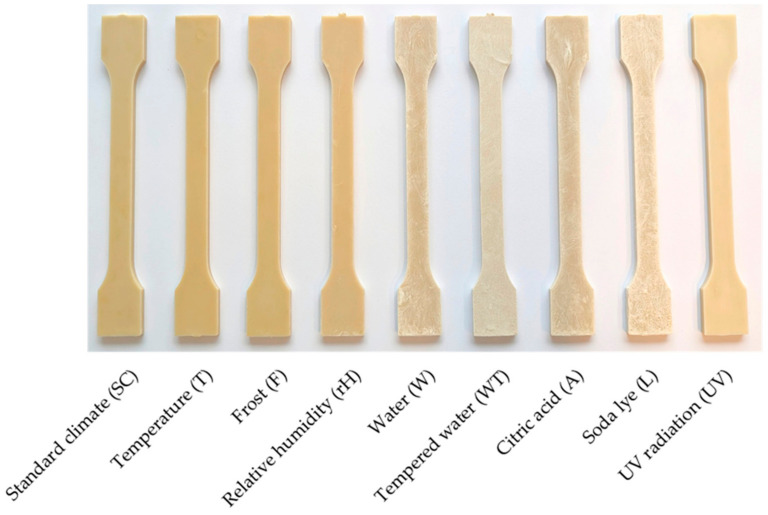
Discoloration of the PHBV-RCF20 test specimens after different conditions of accelerated aging.

The results of the color measurements are described below. The discolorations of the compounds after the various storage conditions are shown in Figure 4 for one test specimen in each case. The color of the test specimens has changed the least after the storage conditions T, F and rH compared to the standard climate. UV radiation causes a brightening of the specimen and after the storage conditions W, WT, A and L the compounds are strongly bleached.

**Figure 5:**
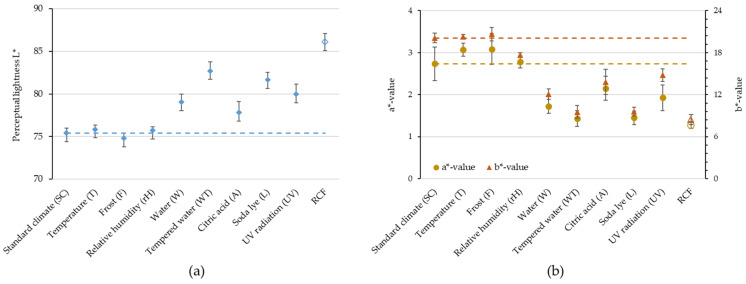
Results of the color tests in the CIELAB color space. Brightness (**a**) and color (**b**) of the PHBV-RCF20 compounds after different storage conditions and the values of the RCFs. Dashed lines symbolize the deviation from the results in standard climate.

These qualitative results are also confirmed by the color measurements. Figure 5 shows the values for brightness (a) and color (b). After standard climate storage, the test specimens already have a light color with an L* value of 75.4. The L* value of a pure PHBV was measured at 74.61 (SD = 0.39) and that of the RCFs at 86.09 (SD = 0.97). This indicates that the PHBV matrix envelops the RCFs and the specimen surface is covered by the polymer.

**Figure 6:**
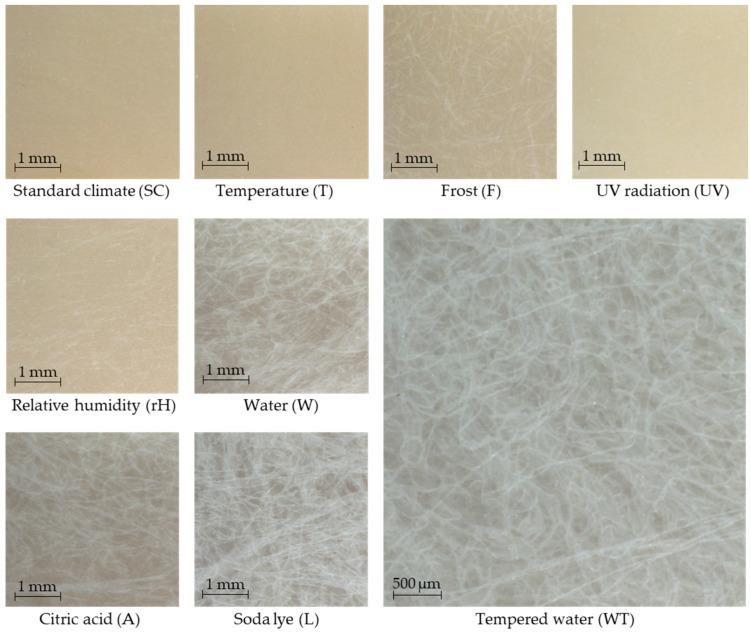
Macroscopic images of the PHBV-RCF20 test specimen surfaces after different storage conditions.

The results of the storage conditions T, rH and F are within the standard deviation of standard climate storage. It is noticeable that storage in water or aqueous solutions (W, WT, A and L) and the influence of UV radiation resulted in significant fading. This applies most strongly to the WT and L storages. The results of the color values a* and b* are analogous to those of the brightness. Here too, the greatest deviations from the normal climate were found in the media and storage conditions under UV radiation. With these storing conditions, the L*, a* and b* values approach those of the RCFs. The reason for this can be seen in the macroscopic images in Figure 6.

While the brightening after UV storage is caused by the fading of the polymer, the RCFs on the specimen surfaces are clearly visible after W, A, L and WT. The PHBV was, therefore, washed out or degraded during all storages in a medium. This surface erosion during the hydrolytic degradation of PHBV has also been described in other studies [2,39,40] and has now also been visually verified here due to the exposed fibers.

### 3.3. Weight Measurements and Rheological Tests

**Figure 7:**
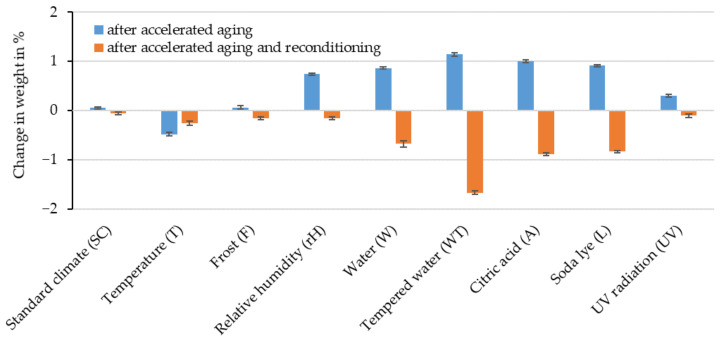
Percentage deviation in the weight of the PHBV-RCF20 test specimens after accelerated aging and after reconditioning in relation to the weight before accelerated aging.

The weight of the test specimens was measured before and after storage and after reconditioning in a standard climate for 21 days. The percentage changes in weight due to storage (blue) and reconditioning (orange) in relation to the weight before storage can be seen in Figure 7.

No mass degradation was observed on the test specimens that were stored in a standard climate for the entire time. In general, it was found that the PHBV-RCF compounds absorb little water over the 21-day period of accelerated aging despite the high water absorption of RCFs. The test specimen mass increased by around 1% each after W, WT, A and L. At a relative humidity of 90%, the change in mass was 0.74%. T resulted in a 0.5% reduction in mass due to the desorption of the water previously present. The initial mass is no longer reached even after reconditioning, which could indicate age-related degradation. After reconditioning the previously stored test specimens, a remaining mass reduction of between 0.68% and 1.67% was observed after storage in water or in aqueous solutions (W, A, L and especially WT). This reduction is not only due to the desorption of the water but also to the hydrolytic degradation of the polymer chains, which is accelerated by the increased temperature during storage in WT. These results correspond with the previously described results of the macroscopy tests, in which a surface erosion of the PHBV and an exposure of the RCFs was observed after W, WT, A and L.

**Figure 8:**
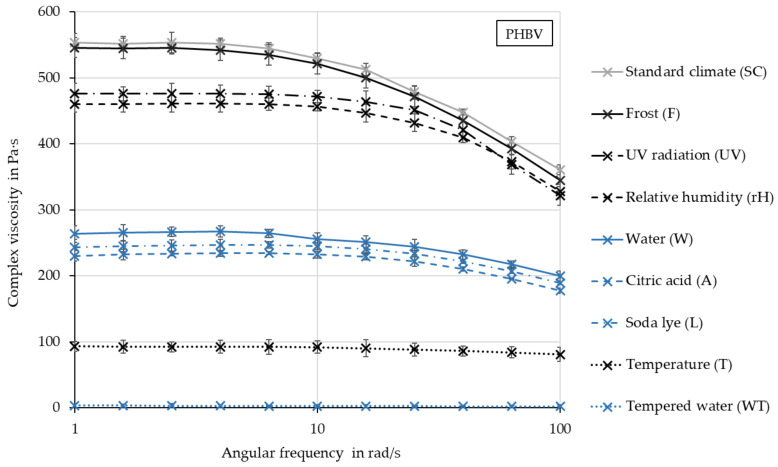
Viscosity of pure PHBV after various accelerated aging tests.

In order to detect possible polymer chain degradation, samples of pure PHBV were aged and then tests on a plate–plate rheometer were carried out. Figure 8 shows the complex viscosity of the samples after various weathering tests as a function of the angular frequency. At an angular frequency of 1 rad/s, the complex viscosity after SC is 553.7 Pa s. After all other storages, the viscosity is lower. After storage F, the viscosity decreases the least, whereas it is slightly reduced after UV and rH. A more significant reduction in viscosity results after the aging conditions W, A and L, whereby the drop after W is slightly lower. A decrease in viscosity of 93.25% was observed after T. Since storage T was carried out at 50% relative humidity, thermal or hygrothermal degradation is suspected here, which, as the macroscopic images of the compounds show, does not originate exclusively from the test specimen surface. After storage in water at 70 °C, the sample had such a low viscosity (3.93 Pa s at 1 rad/s) that measurements on the rheometer proved to be very difficult. The corresponding compounds at WT showed a pronounced erosion of the polymer from the surface. The extremely low viscosity of the pure PHBV also indicates a high degree of aging via hydrolysis inside the test specimens, which was catalyzed via the higher temperature.

### 3.4. Mechanical Testing

**Figure 9:**
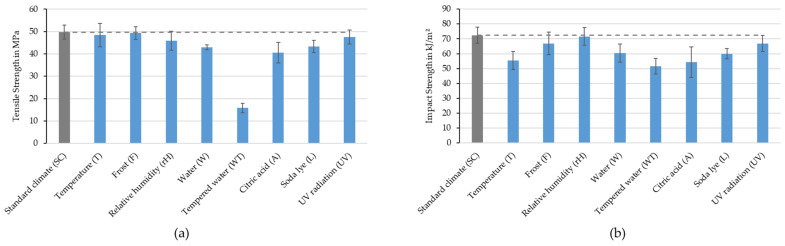
Tensile strength (**a**) and impact strength (**b**) of PHBV-RCF compounds with 20 wt.% fiber content after different storage conditions. Dashed lines symbolize the deviation from the results in standard climate.

The effect of the detected aging mechanisms on the mechanical properties was investigated using tensile tests and impact tests after artificial weathering. Figure 9 shows the resulting tensile strengths (a) and impact strengths (b) of the weathered compounds in relation to the unweathered compounds (SC). 

There are no significant changes in the tensile strengths after the T, F and UV. Even though the viscosity of the pure PHBV after T was greatly reduced through the degradation of the polymer chains, this had no effect on the tensile strength of the compounds. rH, W, A and L resulted in a slight reduction and storage WT resulted in a very strong reduction in tensile strength of 69.49% compared to SC. Additional tests on pure PHBV showed a reduction in tensile strength from 37.82 MPa to 6.32 MPa (−83.32%) after WT storage and, thus, an even greater reduction than with the RCF compounds. It should be noted here that all compounds in which the tensile strength was significantly reduced were exposed to water or an aqueous solution. As previously explained, hydrolytic degradation is assumed to be the cause of this. This degradation is catalyzed by the increased temperature at 70 °C during WT storage.

In general, the reduction in impact strength after accelerated aging is less than the reduction in tensile strength. The impact strength of the compounds remains at the level of the standard climate after the rH storage conditions and is within the standard deviation after frost storage. After UV storage, the tensile strength is slightly decreased. As with the tensile strength, W, A, L and especially WT (−28.68%) reduced the impact strength the most. But in contrast, a significant decrease of 23.61% in the impact strength can be seen after temperature storage, which can be attributed to thermal or hygrothermal degradation, as also indicated via the rheological investigations.

## 4. Conclusions

PHBV-RCF compounds with good strength and very high impact strength can be produced. Due to the improved mechanical properties, the compounds can meet the requirements of more highly stressed components.

The accelerated aging tests carried out on the PHBV-RCF20 compounds showed varying degrees of degradation depending on the type of accelerated aging. 

After storage in water and in aqueous solutions (W, L, A and WT), the test specimens were significantly brighter. After standard climate storage, the brightness values of the pure PHBV and the PHBV-RCF compounds were at a comparable level. These results and the macroscopy images suggest that the PHBV matrix completely encloses the RCFs after standard climate storage and that there are almost no fibers on the test specimen surface. After accelerated aging in media, the brightness values approached those of the RCFs. In macroscopic images, an erosion of the PHBV matrix from the compound surface could be detected as the reason for this. The surface erosion led to a permanent reduction in the test specimen mass of up to 1.67% after water storage at elevated temperatures. The mechanical properties of the compounds, more precisely the tensile strength and impact strength, were also significantly reduced after storage in water or aqueous solutions. This was most pronounced in the tensile strength after storage in WT with a reduction of 68.24%. The corresponding very low viscosities of pure PHBV again indicate a significant shortening of the polymer chain due to thermally catalyzed hydrolytic degradation, which takes place not only on the surface but also inside the compounds.

After temperature storage, the test specimens hardly changed optically, which was reflected in the measured color values and macroscopic images. The viscosities of the pure PHBV were greatly reduced after T, which also indicates thermal or hygrothermal degradation. This degradation had no effect on the tensile strength, but the impact strength was 23.61% lower than after standard climate storage. In the future, further accelerated aging tests such as temperature storage in a vacuum to determine which degradation processes dominate and extended characterization with FTIR or GPC should be carried out.

The influence of UV radiation led to a brightening of the compounds, which, in contrast to the media storage, was not due to the exposure of fibers. No fibers were visible on the test specimen surface in the macroscopic images. The brightening could rather be due to photo-oxidative aging processes. These processes do not lead to a reduction in the mechanical properties in the tensile and impact tests carried out, as photochemical processes initially only originate from the test specimen surface. However, the formation of radicals due to the breakage of polymer chains can initiate further degradation processes over time in combination with other environmental influences. Such combined degradation mechanisms should be investigated in further studies.

The use of RCFs has significantly increased the mechanical properties of PHBV, making it suitable for use in short-life applications with higher loads. For use in longer-lasting products, the stabilization of the compounds is conceivable through the use of additives that prevent degradation.

## Figures and Tables

**Table 1:** Conditions of the accelerated aging tests.

Accelerated Aging	Media	Temperature in °C	UV Radiation Intensity in W/m^2^	Relative Humidity in %
Standard Climate (SC)	-	23	-	50
Temperature (T)	-	70	-	50
Frost (F)	-	−18	-	-
Relative Humidity (RH)	-	23	-	90
Water (W)	Water (pH = 7)	23	-	-
Tempered Water (WT)	Water (pH = 7)	70	-	-
Acid (A)	Citric acid (pH = 2)	23	-	-
Lye (L)	Soda lye (pH = 12)	23	-	-
UV Radiation (UV)	-	23	1000	50

Parameters marked in green were actively set to the conditions of the standard climate for easier comparability of the results.

## Data Availability

The data collected in this study can be obtained upon request from the corresponding author. The data are not publicly available due to the large amount of data.

## References

[1] Kuciel S., Mazur K., Jakubowska P. (2019). Novel Biorenewable Composites Based on Poly(3-hydroxybutyrate-co-3-hydroxyvalerate) with Natural Fillers. J. Polym. Environ..

[2] Lyshtva P., Voronova V., Barbir J., Leal Filho W., Kröger S.D., Witt G., Miksch L., Sabowski R., Gutow L., Frank C. (2024). Degradation of a poly(3-hydroxybutyrate-co-3-hydroxyvalerate) (PHBV) compound in different environments. Heliyon.

[3] Kim J., Gupta N.S., Bezek L.B., Linn J., Bejagam K.K., Banerjee S., Dumont J.H., Nam S.Y., Kang H.W., Park C.H. (2023). Biodegradation Studies of Polyhydroxybutyrate and Polyhydroxybutyrate-co-Polyhydroxyvalerate Films in Soil. Int. J. Mol. Sci..

[4] Rivera-Briso A.L., Serrano-Aroca Á. (2018). Poly(3-Hydroxybutyrate-co-3-Hydroxyvalerate): Enhancement Strategies for Advanced Applications. Polymers.

[5] Antunes A., Popelka A., Aljarod O., Hassan M.K., Kasak P., Luyt A.S. (2020). Accelerated Weathering Effects on Poly(3-hydroxybutyrate-co-3-hydroxyvalerate) (PHBV) and PHBV/TiO_2_ Nanocomposites. Polymers.

[6] Sánchez-Safont E.L., Arrillaga A., Anakabe J., Gamez-Perez J., Cabedo L. (2019). PHBV/TPU/cellulose compounds for compostable injection molded parts with improved thermal and mechanical performance. J. Appl. Polym. Sci..

[7] Rodríguez-Cendal A.I., Gómez-Seoane I., de Toro-Santos F.J., Fuentes-Boquete I.M., Señarís-Rodríguez J., Díaz-Prado S.M. (2023). Biomedical Applications of the Biopolymer Poly(3-hydroxybutyrate-co-3-hydroxyvalerate) (PHBV): Drug Encapsulation and Scaffold Fabrication. Int. J. Mol. Sci..

[8] Weng Y.-X., Wang X.-L., Wang Y.-Z. (2011). Biodegradation behavior of PHAs with different chemical structures under controlled composting conditions. Polym. Test..

[9] Wei L., McDonald A.G. (2016). Accelerated weathering studies on the bioplastic, poly(3-hydroxybutyrate-co-3-hydroxyvalerate). Polym. Degrad. Stab..

[10] Xiang H., Wen X., Miu X., Li Y., Zhou Z., Zhu M. (2016). Thermal depolymerization mechanisms of poly(3-hydroxybutyrate-co-3-hydroxyvalerate). Prog. Nat. Sci. Mater. Int..

[11] Srubar W.V., Wright Z.C., Tsui A., Michel A.T., Billington S.L., Frank C.W. (2012). Characterizing the effects of ambient aging on the mechanical and physical properties of two commercially available bacterial thermoplastics. Polym. Degrad. Stab..

[12] Iggui K., Kaci M., Le Moigne N., Bergeret A. (2021). Effects of hygrothermal aging on chemical, physical, and mechanical properties of poly(3-hydroxybutyrate-co-3-hydroxyvalerate)/Cloisite 30B bionanocomposite. Polym. Compos..

[13] Ibrahim M.I., Alsafadi D., Alamry K.A., Hussein M.A. (2021). Properties and Applications of Poly(3-hydroxybutyrate-co-3-hydroxyvalerate) Biocomposites. J. Polym. Environ..

[14] Mazur K.E., Jakubowska P., Gaweł A., Kuciel S. (2022). Mechanical, thermal and hydrodegradation behavior of poly(3-hydroxybutyrate-co-3-hydroxyvalerate) (PHBV) composites with agricultural fibers as reinforcing fillers. Sustain. Mater. Technol..

[15] Qiang T., Wang J., Wolcott M.P. (2018). Facile Fabrication of 100% Bio-based and Degradable Ternary Cellulose/PHBV/PLA Composites. Materials.

[16] Sánchez-Safont E.L., Aldureid A., Lagarón J.M., Cabedo L., Gámez-Pérez J. (2020). Study of the Compatibilization Effect of Different Reactive Agents in PHB/Natural Fiber-Based Composites. Polymers.

[17] Erdmann J. (2017). Biobasierte Kunststoffe mit Cellulosefaserverstärkung: Zusammenhänge zwischen Struktur, Haftung und mechanischen Eigenschaften. Ph.D. Thesis.

[18] Feldmann M. (2016). The effects of the injection moulding temperature on the mechanical properties and morphology of polypropylene man-made cellulose fibre composites. Compos. Part A Appl. Sci. Manuf..

[19] Jiang G., Huang W., Li L., Wang X., Pang F., Zhang Y., Wang H. (2012). Structure and properties of regenerated cellulose fibers from different technology processes. Carbohydr. Polym..

[20] Veit D. (2023). Fasern.

[21] Zarges J.-C., Minkley D., Feldmann M., Heim H.-P. (2017). Fracture toughness of injection molded, man-made cellulose fiber reinforced polypropylene. Compos. Part A Appl. Sci. Manuf..

[22] Bledzki A.K., Franciszczak P., Meljon A. (2015). High performance hybrid PP and PLA biocomposites reinforced with short man-made cellulose fibres and softwood flour. Compos. Part A Appl. Sci. Manuf..

[23] Fink H.-P., Ganster J. (2006). Novel Thermoplastic Composites from Commodity Polymers and Man-Made Cellulose Fibers. Macromol. Symp..

[24] Ganster J., Fink H.-P., Uihlein K., Zimmerer B. (2008). Cellulose man-made fibre reinforced polypropylene—Correlations between fibre and composite properties. Cellulose.

[25] Khan M.A., Ganster J., Fink H.-P. (2009). Hybrid composites of jute and man-made cellulose fibers with polypropylene by injection moulding. Compos. Part A Appl. Sci. Manuf..

[26] Graupner N., Müssig J. (2009). Man-Made Cellulose Fibres as Reinforcement for Poly(lactic acid) (PLA) Composites. J. Biobased Mater. Bioenergy.

[27] Bledzki A.K., Jaszkiewicz A., Scherzer D. (2009). Mechanical properties of PLA composites with man-made cellulose and abaca fibres. Compos. Part A Appl. Sci. Manuf..

[28] Graupner N. (2009). Improvement of the Mechanical Properties of Biodegradable Hemp Fiber Reinforced Poly(lactic acid) (PLA) Composites by the Admixture of Man-made Cellulose Fibers. J. Compos. Mater..

[29] Graupner N., Herrmann A.S., Müssig J. (2009). Natural and man-made cellulose fibre-reinforced poly(lactic acid) (PLA) composites: An overview about mechanical characteristics and application areas. Compos. Part A Appl. Sci. Manuf..

[30] Falkenreck C.K., Gemmeke N., Zarges J.-C., Heim H.-P. (2023). Influence of Accelerated Aging on the Fiber-Matrix Adhesion of Regenerated Cellulose Fiber-Reinforced Bio-Polyamide. Polymers.

[31] Feldmann M., Heim H.-P., Zarges J.-C. (2016). Influence of the process parameters on the mechanical properties of engineering biocomposites using a twin-screw extruder. Compos. Part A Appl. Sci. Manuf..

[32] Feldmann M., Bledzki A.K. (2014). Bio-based polyamides reinforced with cellulosic fibres—Processing and properties. Compos. Sci. Technol..

[33] Bledzki A.K., Jaszkiewicz A. (2010). Mechanical performance of biocomposites based on PLA and PHBV reinforced with natural fibres—A comparative study to PP. Compos. Sci. Technol..

[34] Chan C.M., Vandi L.-J., Pratt S., Halley P., Richardson D., Werker A., Laycock B. (2018). Mechanical performance and long-term indoor stability of polyhydroxyalkanoate (PHA)-based wood plastic composites (WPCs) modified by non-reactive additives. Eur. Polym. J..

[35] (2019). Kunststoffe_-Vielzweckprobekörper (ISO_3167:2014).

[36] (2019). Kunststoffe_-Bestimmung der Zugeigenschaften—Teil 1: Allgemeine Grundsätze (ISO_527-1:2019).

[37] (2010). Kunststoffe Bestimmung der Charpy-Schlageigenschaften—Teil 1: Nicht instrumentierte Schlagzähigkeitsprüfung (ISO_179-1:2010).

[38] Zarges J.-C., Sälzer P., Heim H.-P. (2020). Correlation of fiber orientation and fiber-matrix-interaction of injection-molded polypropylene cellulose fiber composites. Compos. Part A Appl. Sci. Manuf..

[39] Deroiné M., Le Duigou A., Corre Y.-M., Le Gac P.-Y., Davies P., César G., Bruzaud S. (2014). Accelerated ageing and lifetime prediction of poly(3-hydroxybutyrate-co-3-hydroxyvalerate) in distilled water. Polym. Test..

[40] Buzarovska A., Grozdanov A., Avella M., Gentile G., Errico M. (2009). Poly(hydroxybutyrate-co-hydroxyvalerate)/titanium dioxide nanocomposites: A degradation study. J. Appl. Polym. Sci..

